# Sex Differences in Obesity Associated with Total Fertility Rate

**DOI:** 10.1371/journal.pone.0010587

**Published:** 2010-05-12

**Authors:** Robert Brooks, Alexei Maklakov

**Affiliations:** 1 Evolution & Ecology Research Centre, School of Biological, Earth and Environmental Sciences, The University of New South Wales, Sydney, New South Wales, Australia; 2 Department of Animal Ecology, Evolutionary Biology Centre, Uppsala University, Uppsala, Sweden; University of Zurich, Switzerland

## Abstract

The identification of biological and ecological factors that contribute to obesity may help in combating the spreading obesity crisis. Sex differences in obesity rates are particularly poorly understood. Here we show that the strong female bias in obesity in many countries is associated with high total fertility rate, which is well known to be correlated with factors such as low average income, infant mortality and female education. We also document effects of reduced access to contraception and increased inequality of income among households on obesity rates. These results are consistent with studies that implicate reproduction as a risk factor for obesity in women and that suggest the effects of reproduction interact with socioeconomic and educational factors. We discuss our results in the light of recent research in dietary ecology and the suggestion that insulin resistance during pregnancy is due to historic adaptation to protect the developing foetus during famine. Increased access to contraception and education in countries with high total fertility rate might have the additional benefit of reducing the rates of obesity in women.

## Introduction

Recently the number of obese people on earth exceeded for the first time the number of people who do not get enough to eat [1]. Because the obesity crisis is most dramatic in nations that have escaped from hunger, it is tempting to think of obesity as a consequence of wealth, affecting people who can afford excess food, who do not walk long distances or do physical labour. Within developed nations, however, obesity is strongly associated with socioeconomic disadvantage [2], [3], [4], [5], and low socioeconomic and educational status seem to have particularly strong effects on obesity rates among women [6], [7], [8].

There are enormous differences among countries in obesity rates, from less than one percent of adults in Ethiopia and Cambodia to more than sixty percent of adults in Nauru and the Cook Islands [9]. Much of this variation is associated with differences among countries in economic development and associated phenomena like medical care, urbanisation, education, leisure time and sedentary work. Although the risk of obesity is known to be mediated by sex [5], [10], [11], [12] it is less commonly noted just how different rates of obesity are between men and women in different countries. Only a very few countries have higher levels of male than female obesity, and where there are large disparities between men and women in obesity, far more women are obese than men. As far as we are aware there are no published studies that attempt to explain why countries differ in the size of the male-female obesity gap.

Variation in and relationships among life history traits such as lifespan, reproductive effort and weight gain can be understood by studying them at a variety of scales, from longitudinal studies on individual subjects to large international data sets. Although international data are by their nature very coarse in resolution, they do tend to capture a wider range of variation in economic and cultural factors than more focussed experimental, longitudinal or neighbourhood-level studies. As such they are an indispensible tool for identifying the range of phenotypically plastic strategies that humans are capable of, and generating hypotheses for more direct testing. This is particularly true for questions that involve sex differences. For example, Maklakov [13] recently showed that national total fertility rates (mean number of children produced by each woman between ages 15–45 years assuming that current age-specific birth rate remains constant) explain a large proportion of the variation among countries in women's longevity and thus in the difference in lifespan between men and women. In countries where women have few children they tend to live longer than men, but where birth rates are high the sex bias in lifespan is small or even reversed.

The striking pattern of high female obesity relative to male obesity in many nations requires explanation [14]. In this paper, we make a preliminary, correlative attempt to identify possible factors that may contribute to this pattern. To do so, we explore publicly available data on obesity and on indicators of socioeconomic development, demography and the status of women in order to better understand the factors that might be at play in generating such surprising disparities among countries.

## Methods

We used standardised obesity data from the World Health Organisation's Global Database on Body Mass Index (WHO 2010) which includes the results of a large number of surveys and studies. For many countries, male and female obesity rates were available from the same study. Where this was not the case, data for adult men and women were always obtained from samples within 3 years of one another. To avoid confounding effects of temporal trends in obesity, we also only used data from surveys post 1998, and for countries where there were multiple surveys post-1998 we used the most recent. There were suitable female obesity data for 137 countries, but suitable male data for only 94 of these countries. Data represent percentage of adult (older than 15 years) men and women with Body Mass Index greater than or equal to 30.0, the standard WHO definition of adult obesity rate. We used the Central Intelligence Agency World Factbook (www.cia.gov) and Population Reference Bureau (www.prb.org) databases to extract data on national per capita income (GNI PPP), inequality in income among households (the Gini index), population density, percent urbanisation, number of years of education for both females and males, contraception use, total fertility rate, and infant mortality rate. Many of these traits are functionally correlated, and they tend to also show a latitudinal gradient (e.g. more poor countries with high fertility, low education, high infant mortality and low urbanisation near the equator). We therefore also included absolute latitude in our dataset.

We estimated pairwise Pearson's correlations and fitted multiple regressions in JMP 7.0.2. When building a multiple regression to explain female obesity, we fitted male obesity (and vice versa) to represent all of the broad factors such as food availability and national diet that cause obesity in general, and then fitted other variables to explain the sex difference in obesity. We used Mallows *Cp* statistic to fit the most efficient multiple regression model out of all the possible combinations. We confirmed these models using forward stepwise multiple regression.

## Results and Discussion

Although male and female obesity rates are strongly correlated (Table 1), on average five percent more women are obese than men (paired-sample *t_93_* = 7.70, P<0.0001). Adult female obesity was strongly correlated with adult male obesity, latitude, and with all of the measures of national socioeconomic, demographic and reproductive conditions that we analysed, other than population density and the Gini index of household income inequality (Table 1). Interestingly, all of the significant correlations are in the direction we would predict if economic development and national wealth were associated with greater female obesity; high adult female obesity is associated with high male obesity, more northern latitudes, high income, greater urbanisation, more years of education (both sexes) and greater use of contraception as well as low birth rates and low infant mortality rates. These correlations are consistent with obesity being an affliction of wealthy nations in which a large proportion of the population have escaped from hunger and the demographic transition is well into the fertility decline phase.

**Table 1:** Pairwise Pearson's correlations between adult female and male obesity rates and various measures predicted to influence life-history.

	Female obesity		Male obesity	
	r	N	r	N
Male obesity	0.91***	94		
GNI PPP	0.51***	136	0.33*	93
Population density	−0.04	135	−0.10	92
Urbanisation	0.55***	135	0.44***	92
Female education	0.53***	121	0.44***	85
Male education	0.43***	121	0.31*	85
Contraception	0.36***	115	0.06	74
Total Fertility Rate	−0.36***	136	−0.05	93
Infant mortality rate	−0.55***	136	−0.38**	93
Latitude	0.31**	136	0.11	93
Gini Index	−0.039	112	−0.052	75

***P<0.0001

**P<0.001

*P<0.01

**Figure 1:**
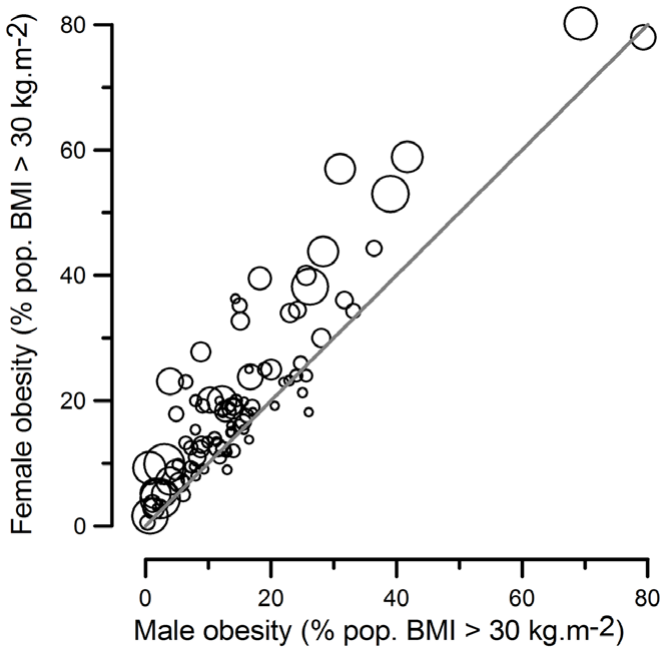
National prevalence of adult obesity in women and men in relation to fertility. Bubble sizes represent Total Fertility Rate.

Multiple regression analysis tells quite a different story. The first and by far the most important predictor of female obesity is male obesity rate which explains 83.0 percent of the variance in female obesity. The best multiple regression model (the smallest model for which *Cp* < the number of parameters) for the larger dataset that did not include Gini (this index was only available for a smaller subset of countries) includes only the intercept, male obesity (*β* = 0.80±0.03 S.E., P<0.001) and total fertility rate (*β* = 0.38±0.08 S.E., P<0.001) (model R^2^_adj_ = 0.86, F_2,90_ = 283.2, P<0.0001). Once the circumstances that influence the general level of adult obesity within countries are controlled for (by fitting male obesity), the statistical effect of total fertility rate on female obesity is positive, as illustrated in Figure 1. This change in sign of the effect of TFR is not an artefact of multicollinearity – the Variance Inflation Factors for male obesity and TFR in this multiple regression were both very low (VIF = 1.05).

The best regression for the smaller subset of data that included Gini includes male obesity, TFR, contraception use and income inequality (R^2^_adj_ = 0.86, F_4,55_ = 90.4, P<0.0001). This model includes far fewer countries because of the number of missing values for the Gini index. Higher female obesity was associated with less contraceptive use (*β* = −0.006±0.003 S.E., P = 0.022) and greater income inequality (*β* = 0.009±0.004 S.E., P = 0.047). Once again all VIF were low (<1.6) suggesting no multicollinearity problems.

In all of the other multiple regression models that we tried in which male obesity was the first term fitted and the second term added was not TFR, the second term we added indicated that obesity was associated with socioeconomic disadvantage and low status of women. This comes about because high total fertility rates, infant mortality, low GNI PPP, high Gini index, few years of female education, low contraception use, and low urbanisation all tend to be correlated as a suite of traits. The associations between these traits are well documented, both within and among countries [15], [16], [17], [18].

Male obesity was strongly correlated with female obesity, but once these effects had been statistically controlled for (*β* = 1.03±0.04 S.E., P<0.001), the only other significant predictor of male obesity was income. GNI PPP had a positive (*β* = 0.22±0.04 S.E., P<0.001) effect on male obesity, in contrast to the female analysis in which GNI PPP would have had a negative effect on female obesity in a model comprising only male obesity and GNI PPP (in such a model the effect of GNI PPP would have been *β* = −0.17±0.04 S.E., P<0.001).

Our results are consistent with smaller-scale studies that document an association between low income, material deprivation, food insecurity or minority status and increased obesity in women but not (or less often) in men [6], [8], [11], [19], [20]. Two influential reviews of the published relationships between socioeconomic status (SES) and obesity [4], [5] indicate that in more economically developed nations, the reported relationships between SES and obesity tend to be negative, but that in less-developed countries (those with low human development index scores) these relationships tend to be positive (i.e. individuals of higher SES tend to be at greater risk of obesity). In both cases the patterns tend to be much stronger and more consistent for women. In men, the fact that national obesity rates are positively associated with high income in our data set suggests that obesity is largely a consequence of a society escaping from hunger and that it is much less strongly mediated by socioeconomic disadvantage than it is in women. Our results suggest that the high incidence of obesity in low SES women from highly developed countries and of higher SES women from less developed countries may be due to a single set of mechanisms. Both of these groups of women are likely to have escaped from chronic hunger unlike the poorest women (and men) in the poorest nations, but they may not have the means to afford or the access to high quality foods that wealthy women in the wealthiest nations can. In order to understand why these effects are much more acute in women, we need to understand how they are mediated by childbearing.

Several recent studies across a variety of countries and circumstances from rural Iraqi women to middle-income Mexicans to Americans of all ethnicities and incomes suggest that parity (the number of times a woman has given birth) is positively associated with increased obesity risk [21], [22], [23], [24], [25], [26]. The relationship between parity and obesity can be modified by socioeconomic and educational circumstances. In the USA, the effects of parity on obesity were greatest in Hispanic and Black women who also tended to score lower in educational and socioeconomic level than White women [20]. A comparison of 28 countries showed that in poorer countries, parity is only or largely associated with obesity among the wealthiest women, but that in wealthier countries parity is associated with greater incidence of obesity across all socioeconomic strata but may be most dramatic among the poor [7].

It appears, therefore, that the role of parity as a trigger for excessive weight gain may be a combined effect of the nutritional and demographic transitions. We predict that the countries with the greatest female bias in adult obesity will be those in which a large proportion of families have escaped from hunger, yet women still have high total fertility rates. If this is true, then access to contraception and the education of women may be just as important in combating obesity as they are in curbing population growth [27]. Our results provide tentative support for the fact that access to contraception, a key index of women's status and wellbeing, reduces the level of female obesity within countries after the effects of parity and male obesity have been statistically controlled for.

Our results also suggest that high income inequality within countries may elevate the incidence of obesity in women but not in men, and that these effects are additional to the effects of parity. The positive contribution of the Gini index to the multiple regression analysis for female obesity suggests that female obesity is governed not only by average wealth, but also by the variation in wealth within societies. Inequality of income is known to be an important determinant of the levels of violence, risky behaviours, accidental death, mental illness, anxiety, and teenage pregnancy within societies [18], [28]. Adult and childhood obesity are also associated with income inequality [18], and our results indicate that the effects of inequality in household income on obesity are particularly strong for women. This effect is consistent with our other findings: societies with high income equality (i.e. low Gini) tend to range from the uniformly poor (e.g Ethiopia: Gini = 30, GNI PPP = $1,190; Albania: Gini = 27, GNI PPP = $5,840) to the uniformly wealthy (e.g. Sweden, Gini = 23, GNI PPP = $34,780; Norway: Gini = 25, GNI PPP = $43,920), whereas high inequality societies tend to be those with mid-range average wealth (e.g. South Africa: Gini = 65, GNI PPP = $11,710, Colombia: Gini = 59, GNI PPP = $7,620). These countries are often in the midst of the demographic and nutritional transitions, with a large proportion of people having escaped from hunger but unable to afford to eat well.

Evolutionary perspectives and large scale correlative studies can play an important role in generating mechanistic hypotheses for health problems like obesity and type 2 diabetes [29], [30], [31]. It is possible that factors causing economic insecurity may have independent effects on total fertility and on obesity in women, generating the patterns that we document here. However, our results are also consistent with a recent proposal that selection since the advent of agriculture may have favoured metabolic traits that put women at elevated risk of type 2 diabetes, cardiovascular disease, polycystic ovary syndrome and diabetes [32]. The historic dependence of agrarian societies on a few key seasonal crops exposed them to seasonal food shortages and occasional famine. Strong fertility selection may have made women, particularly pregnant women, more resistant to insulin, thereby protecting the foetus in times of chronic food shortage. As societies have escaped from the severe periodic food shortages typical of agrarian lifestyles, insulin resistance has begun confer a net fitness disadvantage via the metabolic syndrome and obesity.

Brooks, Simpson and Raubenheimer [33] have recently argued that in developed economies and possibly in less developed economies, the price of protein relative to carbohydrate- and fat-ferived energy may bias poorer consumers toward cheaper high carbohydrate foods and away from dearer high protein foods. Because protein is a powerful regulator of appetite [34], [35], such a bias can result in excessive energy intake and, therefore, obesogenesis [36]. Poorer women in wealthier nations and wealthier women in poorer nations may be particularly vulnerable to this protein leverage effect if they can afford enough high-energy (especially high carbohydrate) foods to become obese, but high costs keep them away from consuming enough protein directly. Such a situation could interact with historic female-specific adaptations to protect the foetus via insulin resistance (e.g., [32]). Wealthy women in wealthy countries and poorer women in poorer countries may be less vulnerable this protein leverage effect because the former are able to afford enough protein and the latter have not yet escaped from hunger (i.e. they do not get enough food to become obese).
